# Can You Trust Your Gut? Implicating a Disrupted Intestinal Microbiome in the Progression of NAFLD/NASH

**DOI:** 10.3389/fendo.2020.592157

**Published:** 2020-10-21

**Authors:** Kavita Jadhav, Taylor S. Cohen

**Affiliations:** Microbiome Discovery, Microbial Sciences, BioPharmaceuticals R&D, AstraZeneca, Gaithersburg, MD, United States

**Keywords:** microbiome, non-alcoholic steatohepatitis, metabolites, gut barrier, gut permeability, non-alcoholic fatty liver disease

## Abstract

Non-alcoholic fatty liver disease (NAFLD) is a spectrum of disorders, ranging from fatty liver to a more insulin resistant, inflammatory and fibrotic state collectively termed non-alcoholic steatohepatitis (NASH). In the United States, 30%–40% of the adult population has fatty liver and 3%–12% has NASH, making it a major public health concern. Consumption of diets high in fat, obesity and Type II diabetes (T2D) are well-established risk factors; however, there is a growing body of literature suggesting a role for the gut microbiome in the development and progression of NAFLD. The gut microbiota is separated from the body by a monolayer of intestinal epithelial cells (IECs) that line the small intestine and colon. The IEC layer is exposed to luminal contents, participates in selective uptake of nutrients and acts as a barrier to passive paracellular permeability of luminal contents through the expression of tight junctions (TJs) between adjacent IECs. A dysbiotic gut microbiome also leads to decreased gut barrier function by disrupting TJs and the gut vascular barrier (GVB), thus exposing the liver to microbial endotoxins. These endotoxins activate hepatic Toll-like receptors (TLRs), further promoting the progression of fatty liver to a more inflammatory and fibrotic NASH phenotype. This review will summarize major findings pertaining to aforementioned gut-liver interactions and its role in the pathophysiology of NAFLD.

## Introduction

Non-alcoholic fatty liver disease (NAFLD), once known as the “un-named” disease, afflicts 80–100 million Americans and is currently the most common cause of chronic liver disease (1). About 20%–30% of NAFLD cases in the United States fall under the more severe category of non-alcoholic steatohepatitis (NASH) (1). With increasing prevalence over the last 20 years, NAFLD presents a burgeoning health problem. Unfortunately no therapies are currently approved for treatment or prevention of NAFLD/NASH. Development of such a therapeutic requires more in depth understanding of this disease, including answers to questions such as: What factors influence progression of steatosis to NASH, to NASH with fibrosis? What predisposes 30% of NAFLD patients to develop NASH? Can we harness pre-disposing factors and other non-invasive methods to accurately predict disease progression?

### Pathophysiology of NAFLD

NAFLD covers a wide range of liver morbidities, with accumulation of lipid droplets being its mildest manifestation, and liver failure or cirrhosis being the worst. When the accumulation of lipid droplets exceeds 5% of the total liver weight, an individual may be characterized as having fatty liver, hepatic steatosis, or non-alcoholic fatty liver (NAFL) (1). About 30% of individuals with NAFL progress to NASH which is characterized by inflammation in addition to lipid accumulation (2). About 20% of NASH patients with advanced fibrosis will progress to cirrhosis, which marks an irreversible decline in liver function, in addition to being a risk factor for hepatocellular carcinoma (2).

**Figure 1:**
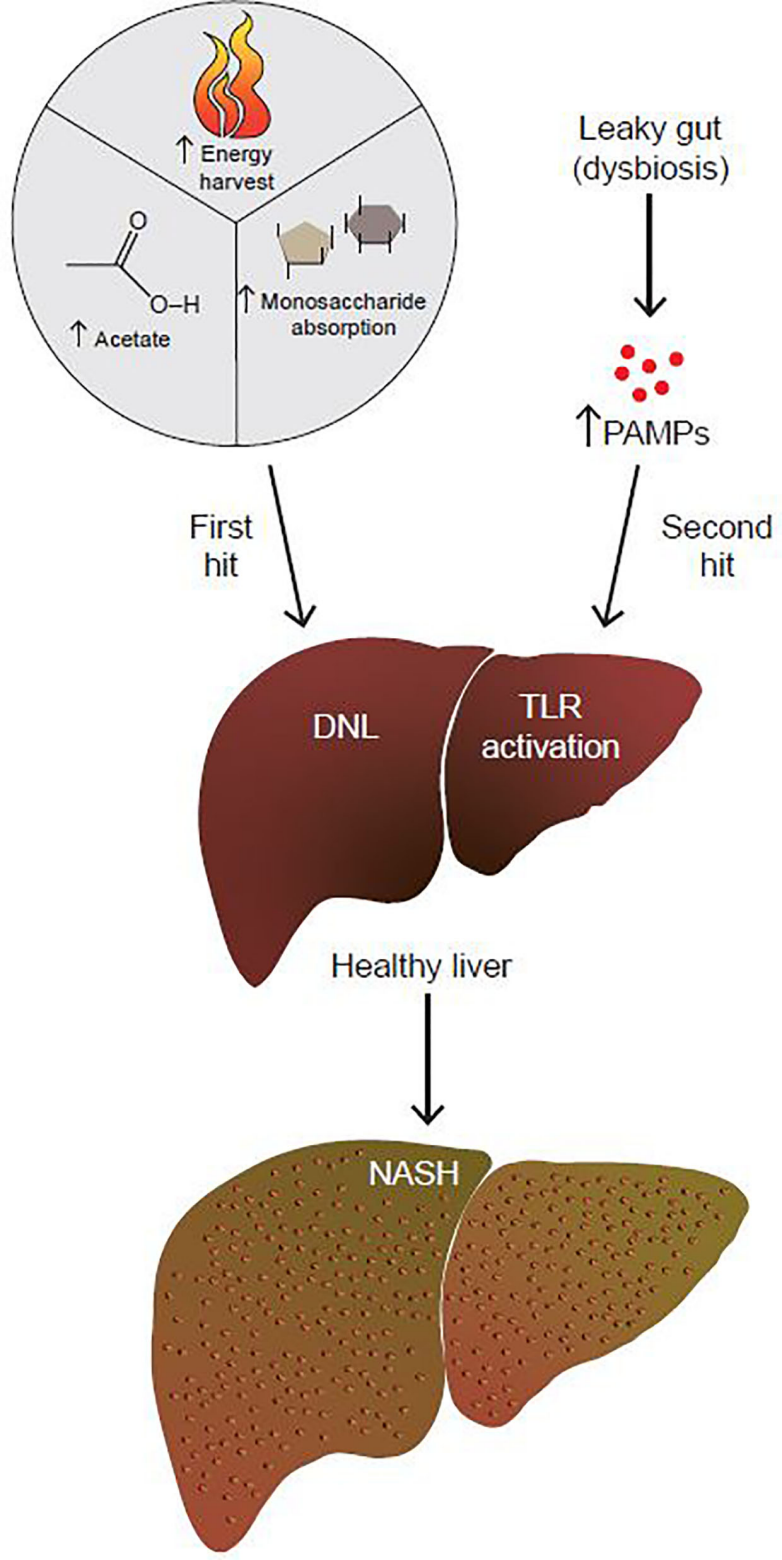
The gut microbiome contributes to both the first and second hits of NAFLD. By increasing energy harvest, monosaccharide absorption, and acetate production, the gut microbiome contributes to the first hit of NAFLD, which is the development of fatty liver. In addition to that, a dysbiotic leaky gut allows for increased passage of PAMPs to the liver. PAMPs activate hepatic TLRs to up-regulate pro-inflammatory and fibrotic pathways. By promoting the development of fatty liver to NASH, the gut microbiome contributes to the second hit of NAFLD.

NAFLD/NASH progression is hypothesized to be due to the combination of insults, termed the two-hit hypothesis (**Figure 1**) (3). This theory postulates that the “first hit” is the development of fatty liver. The “second hit” is characterized by a multitude of factors including inflammatory cytokines, oxidative stress and/or insulin resistance (IR), although the sequence of these events is unclear (3). Since a two-hit model did not sufficiently explain the complex pathophysiology of NAFLD, a more inclusive theory was proposed, the “multiple hit theory” (4). Fatty liver still remains the first hit, but the complex secondary insults reflect broader metabolic dysfunction that involves crosstalk with other organs central to metabolism such as adipose tissue, pancreas, and gut microbiota (4). However, the multiple hit model is still an oversimplification, and additional factors have yet to be fully explored, with age, obesity, and genetic pre-disposition being just a few of them.

On top of a complex etiology, tracking progression of NAFLD is an additional challenge. Serum ALT and fibrosis score are surrogate markers to determine liver damage; however, liver biopsy remains the gold standard for diagnosing and characterizing the different stages of NASH (5). Less invasive alternatives, such as ultrasonography and MRI, allow for visualization of fatty liver, but do not evaluate inflammation or accurately assess fibrosis (6). Limited functionality makes these techniques less viable alternatives as disease development/progression indicators. To complement imaging, biomarker research is an active area of the NAFLD/NASH field. The gut microbiome composition or associated metabolites could be one such biomarker, although additional research is needed to confirm the utility of these approaches.

### Microbiome and Human NAFLD

Fecal microbiota transplant (FMT) studies have implicated the microbiome and NAFLD development in mice (7, 8). However, no particular microbial signature has emerged in human NAFLD, making it difficult to trace disease development back to any particular cluster of bacterial taxa. Sharpton et al. reviewed the reasons behind discordant results obtained from studies trying to draw correlations between the microbiome and human NAFLD (9). A number of confounders could underlie why no one signature has emerged across multiple studies, including differences in patient age, presence of other metabolic co-morbidities, and geographic location. Differences in handling of stool samples, sequencing and statistical analyses performed also could skew the results of individual studies (9). Additionally, compared to 16S ribosomal RNA sequencing, metagenomic analysis allows for a better understanding of the functional and metabolic potential of the gut microbiome (9).

Despite heterogeneity in specific taxa associated with disease, several cross-sectional studies have shown associations between an unhealthy change in the normal bacterial ecology, also known as dysbiosis, and all stages of NAFLD, including fatty liver, NASH, advanced fibrosis and also cirrhosis and hepatocellular carcinoma (10). Aron-Wisnewsky et al. divided human studies into steatosis to NASH, and NAFLD fibrosis to NASH cirrhosis signatures. In doing so, the authors found significant overlap in microbial signatures in both simple steatosis and NASH (10). In brief, steatosis and NASH patients have increased abundance of *Proteobacteria* (13.5%) at the phylum level, increased *Enterobacteriaceae* (12.02%) and decreased *Rikenellaceae (*0.41% in NASH versus 1.97% in healthy patients) and *Ruminococcaceae* (7.01% in NASH versus 18.82% in healthy patients) at the family levels, and increased *Escherichia* (2.36% versus 0.3% in healthy patients), *Peptoniphilus* (4.1% versus 0.36% in healthy patients) and decreased *Anaerosporobacter* (1.08% versus 2.02% in healthy patients), *Coprococcus* (1.03% versus 3.69% in healthy patients), *Eubacterium* (0.29% versus 1.18% in healthy patients), *Faecalibacterium* (4.27% versus 8.15% in healthy patients) and more discordant changes in *Prevotella* at the genera level (6, 11–24). The authors did acknowledge that despite these differences there is widespread divergence in the literature across all levels of taxonomy, with some studies even reporting trends opposite to the ones discussed above (10).

In contrast to simple steatosis, identification of microbial signatures in NASH with fibrosis is less well established, in part due to differences in the threshold for “fibrosis” between studies. For example, some human fibrosis studies have made comparisons between mild to moderate (F0-F2), versus severe fibrosis (F3-F4), while some others have compared no to little fibrosis (F0-F1) to moderate and severe fibrosis (F2-F4), which has created discrepancies in the literature (15, 17). Even then, microbial signatures associated with advanced fibrosis have emerged. In general, advanced fibrosis correlated with increased Gram-negative bacteria, increased *Fusobacteria* phylum, and decreased *Enterobacteriaceae* family and Gram-positive bacteria, *Firmicutes* phylum, *Prevotellaceae* family, and *Prevotella* genus (15, 17, 20). One of these studies utilized metagenomic sequencing along with serum metabolomics which allowed the authors to overlay bacterial abundance with pathway and metabolite enrichment data. This approach provided a more holistic microbial profile of patients with mild/moderate fibrosis, and severe fibrosis with NASH (17). While the gut microbiome signature was consistent with previous studies, serum metabolite analysis revealed increased nucleoside metabolism in severe fibrosis and increased amino acid and carbon metabolism related metabolites in mild/moderate fibrosis (16). In terms of pathway enrichment, mild/moderate fibrosis stool samples were enriched in nucleotide and steroid degradation pathways, while severe fibrosis stool samples were enriched in carbon metabolism and detoxification pathways (19). These data suggest the possibility of harnessing the microbiome to differentiate mild/moderate fibrosis from severe fibrosis with NASH. More studies with the same study design and larger cohort sizes are needed to confirm whether these microbiome-derived signatures can truly be used as a diagnostic tool.

## Role of the Gut Microbiome in the Development and Progression of NAFLD

### Changes in the Gut Microbiome Promote the Development of Fatty Liver

#### Microbiota and Energy Harvest

The human diet is enriched in all three macronutrients, carbohydrates, protein and fat, with carbohydrates making up a bulk of the standard diet. Dietary carbohydrates come in three forms, polysaccharides, disaccharides, and monosaccharides, as defined by the number of monomeric units. In order to be used as energy sources by the host, poly- and disaccharides must first be broken down to their monosaccharide units. Of all the enzymes required for this hydrolysis to occur, humans only encode amylase which removes monosaccharide units from starch. Other than amylase, the host depends on the gut microbiome to harvest energy from dietary polysaccharides (25). Non-starch polysaccharides such as cellulose or hemicellulose are metabolized by colonic bacteria to generate short chain fatty acids (SCFAs) such as butyrate, acetate, and propionate (25, 26). Analysis of feces originating from germ-free (GF) mice revealed significantly reduced levels of SCFAs in the intestine and cecum when compared to conventional mice, supporting the need for commensal bacteria to metabolize non-digestible carbohydrates to generate SCFAs (27). Microbial-produced monosaccharides and SCFAs are absorbed into the portal vein and serve as substrates for *de novo* lipogenesis (DNL) in the liver. So far, 130 families of glycoside hydrolases, 22 families of polysaccharide lyases and 16 families of carbohydrate esterases have been discovered, and a vast majority of these are encoded in microbial genomes (28). In addition, metagenomic sequencing of human gut microbiota has uncovered a vast panel of carbohydrate-active enzymes (CAZymes) including hydrolases, lyases and esterases, a great majority of which remain to be characterized (25).

**Figure 2:**
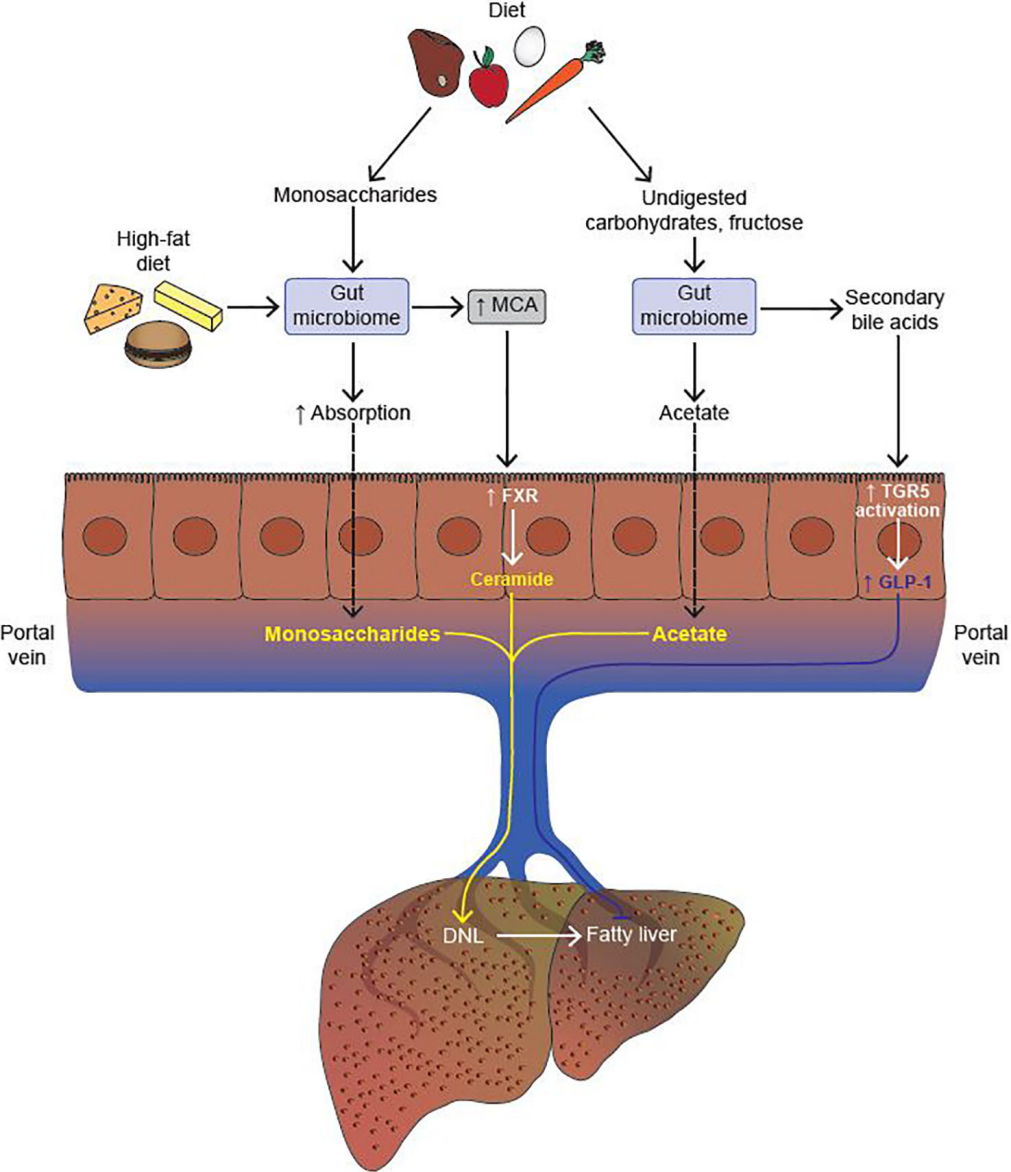
The gut microbiome modulates the development of fatty liver. The gut microbiome increases absorption of monosaccharides from the diet, thereby promoting hepatic *de novo* lipogenesis (DNL) by increasing substrate availability. Upon consumption of a high fat diet, the gut microbiome increases the production of muricholic acid (MCA) in mice. MCA is a potent activator of intestinal farnesoid X-receptor (FXR), which, in turn, activates the ceramide synthesis pathway in intestinal epithelial cells (IECs). Upon reaching the liver, ceramide promotes the cleavage of Srebp-1c, and thus upregulation of the hepatic DNL program. Undigested carbohydrates and fructose are processed by the gut microbiome to generate acetate, a substrate for hepatic DNL. Lastly, secondary bile acids (BAs) generated by the gut microbiome activate the Takeda G protein-coupled receptor (TGR5) expressed in colonic L cells which, in turn, increases the secretion of glucagon-like peptide 1 (GLP-1). GLP-1 inhibits the development of fatty liver both by driving down DNL, and by increasing fatty acid oxidation in hepatocytes (Activation, indicated in yellow; Inhibition, indicated in blue).

In addition to SCFA generation, another mechanism by which the gut microbiome contributes to energy harvest is by increasing the absorption of dietary monosaccharides across the intestine (29). Conventionally housed mice that were given an oral bolus of glucose showed twice the monosaccharide absorption across the intestine as compared to GF mice (29). Absorbed monosaccharides were then transferred to the portal vein, thereby increasing substrate availability for hepatic DNL (**Figure 2**).

Perhaps, the most direct link between energy harvest, availability of SCFAs and hepatic steatosis was provided in a study that investigated the role of GPR41, a receptor for acetate and propionate (30). Through bomb-calorimetric assays of feces, this study demonstrated that the efficiency of caloric extraction from a polysaccharide rich chow diet was significantly reduced in Gpr41-deficient versus wild-type mice, although the mechanism behind this was unclear. In addition, cecal levels of acetate and propionate were significantly increased in the knockouts, indicating increased excretion of SCFAs. Concomitantly, hepatic triglycerides (TGs) in the Gpr41-deficient mice were significantly reduced (30). The reason behind this phenotype might be that a lack of Gpr41 prevents uptake of dietary polysaccharide-derived SCFAs. Therefore, energy harvest in the absence of SCFA receptors is deemed redundant as this promotes DNL substrates like SCFAs to be excreted in the feces, thereby reducing hepatic steatosis.

From a translational perspective, there are limited human data on any of these mechanisms in fatty liver development. The literature suggests that obese individuals have increased intestinal glucose absorption, but this has not been tied back to the microbiome (31). Monosaccharide transporters might be potential targets, but selection will be a challenge as the GLUT family of transporters alone has 14 members. The SCFA receptors GPR41 and GPR43 show functional divergence when it comes to differentiation of adipocytes, but whether these differences also apply to their role in SCFA uptake is largely unknown (32). In addition, there are no human data on the expression of these receptors in intestinal epithelial cells (IECs) in metabolic disease. Lastly, enrichment of glycoside hydrolase metagenomic signatures in obese mice and humans unable to lose weight on a lifestyle intervention program holds translational promise, but the functional value of these signatures remains to be identified in humans (33, 34). One way to assess functionality would be to measure if microbiome-derived acetate feeds into hepatic DNL as a result of increased expression of glycoside hydrolase in obesity, but this may not be that straight-forward in humans. One potential “fix” for removing glycoside hydrolase-rich microbial populations could be to re-populate the obese gut with FMT from healthy donors. In fact, there currently are clinical trials using this technique to evaluate its impact in NAFLD (ClinicalTrials.gov Identifier: NCT02469272).

#### Microbiota as a Source of DNL Substrates

Hepatic DNL is the process by which excess carbohydrates in the liver are converted to neutral TGs and stored in lipid droplets. Depending on the energy state of the cell, these TGs are either packaged into very low density lipoprotein (VLDL) particles and secreted out of the liver or are hydrolyzed to undergo β-oxidation. As such, DNL has two components; synthesis of free fatty acids (FFAs) and incorporation of 3 FFAs onto 1 glycerol to form one molecule of tri-acyl glycerol (TAG). Substrates for DNL are sourced from both the host in the form of FFA flux from the adipose tissue, and also microbiome-derived metabolism of carbohydrates and fatty acids from the diet.

Microbial products like SCFAs serve as substrates for hepatic DNL, thereby accelerating the development of fatty liver. Kindt et al. integrated transcriptomic, proteomic, phosphoproteomic, and lipidomic analyses of livers from GF and specific pathogen-free (SPF) mice to provide a comprehensive, multi-OMICS based link between the microbiome and hepatic lipogenesis (35). Presence of microbiota led to a significant increase in desaturation of the FA palmitate by SCD-1, and elongation of the FA γ-linoleic acid by fatty acid elongase (ELOVL)-5. In addition, significant increases were also observed in other TAG-synthesizing enzymes such as fatty acid synthase (FAS), further promoting the development of fatty liver (35). Strikingly, oral gavage of labeled acetate led to its rapid incorporation into newly forming C16 and C18 fatty acids in the livers of SPF mice, further corroborating the idea that SCFAs produced from microbial fermentation of dietary fiber serve as substrates for hepatic DNL (35). While the SCFAs propionate and butyrate have been shown to protect against NAFLD, acetate acts as a substrate for DNL in hepatocytes (36–39) (**Figure 2**). So while acetate can be assigned as pro-lipogenic, the same does not apply to all SCFAs. Having said that, a recent study by Rau et al. drew correlations between SCFAs and NAFLD severity (40). Thirty-two NAFLD patients were further stratified into non-alcoholic fatty liver (NAFL) and NASH patients. Compared to healthy controls (HCs), there was a 50% increase in fecal acetate and a 100% increase in propionate levels in patients with NAFL, while no difference was observed in butyrate levels (40). A similar trend in acetate and propionate levels was observed when NASH patients were further stratified into mild (F0-F1) and moderate/severe (F2-F4) NASH in comparison to HCs. However, patients with mild NASH had modest but statistically significant higher levels of fecal acetate (~20%) and propionate (~25%) compared to patients with moderate/severe NASH. At the family level, the gut microbiome of NAFLD (NAFL/NASH) patients was enriched in *Fusobacteriaceae* and *Prevotellaceae* compared to the gut microbiome of HCs. Both bacterial families are well characterized SCFA-producers, thereby providing a functional link between the microbiome and microbial metabolites (40). There were no differences in bacterial populations at the family level between the mild and moderate/severe NASH groups, but *Acadaminococcus* and *Prevotella* were more enriched in moderate/severe NASH (40).

While the above study failed to establish any causal links in the microbiome-SCFA-NAFLD axis, it highlighted some interesting findings. For one, both fecal acetate and propionate are increased in patients with NAFLD versus HCs, but upon closer observation, it is clear that in the NAFL group, acetate levels are three times greater than propionate levels, even though acetate levels went up by only 50%, while propionate went up by 100% when compared to HCs (40). This could mean that while the two SCFAs have opposing roles in DNL, net higher levels of acetate may tip the balance in favor of a pro-lipogenic phenotype. More interestingly, both acetate and propionate levels drop modestly (around 15%) in moderate/severe NASH (F2-F4) in comparison to mild (F0-F1) NASH, which could mean that while more SCFAs are produced in the early stages of NAFL and NASH, this may not be the case when fibrosis becomes more severe. Since it is hard to predict causality in human studies, some future directions for finding a link between SCFAs and NAFLD could be carbon tracing the metabolism of microbiome-derived acetate in mouse models of fatty liver and NASH. Using oral administration of 13C-acetate, the amount of labeled carbons that are incorporated in end products of DNL can be estimated by mass-spectrometry. Another approach would be dual tracing of acetate and propionate in the same mouse models to get a more complete picture of SCFA metabolism in NAFLD. In addition to carbon tracing, another angle would be examining the effect of SCFAs on inhibition of histone deacetylases (HDACs), as it has been demonstrated that SCFAs like butyrate inhibit HDACs to activatethe transcription of activators of fatty acid oxidation such as peroxisome proliferator-activated receptor (PPAR)-α.

While these data are suggestive that gut microbiome derived metabolites contribute to hepatic DNL, they provide an incomplete picture. DNL is also heavily modulated by Srebp-1c and ChREBP transcriptionally, but there are limited data on relative contribution of the transcriptional DNL program versus gut microbiome (41). Indeed, GF mice are resistant to HFD-induced obesity and fatty liver, indicating that complete ablation of the gut microbiome suppresses the transcriptional DNL program through yet unknown mechanisms (42). Conversely, Srebp-1c and ChREBP knockouts are resistant to NAFLD development even in the presence of the microbiome (43, 44). This implies that the absence of the transcriptional DNL program either changes the microbiome composition such that there is less production of DNL substrates, or microbial lipogenic substrates fail to induce DNL by themselves. Either way, crosstalk between the two, or emergence of alternative metabolic pathways in the absence of one or the other remains to be elucidated.

In their recent paper, Zhao et al. set out to tease apart the relative contribution of these two pathways in fructose consumption-mediated increases in hepatic DNL (45). Counter to current dogma surrounding the role of dietary fructose in hepatic steatosis, Zhao et al. demonstrated that dietary fructose is converted to acetate by the gut microbiome, and can be used by the liver as a precursor for DNL (45). Prior to these data, it was believed that once in the hepatocyte, fructose enters the tricarboxylic acid (TCA) cycle and is converted to citrate. ATP citrate lyase (ACLY) converts citrate to acetyl-Coa which serves as a precursor for DNL. By knocking out ACLY, Zhao et al. demonstrated that dietary fructose can still contribute to the NAFLD phenotype by bacterial conversion to acetate, followed by transport to the liver *via* the portal vein. In the liver, acetate is converted to acetyl-Coa by acetyl-CoA synthetase (ACSS)-2 and is ultimately shunted into the lipogenic pathway. Finally, gene expression of ChREBP-β and other DNL genes is upregulated upon fructose feeding independently of acetyl-CoA metabolism. Collectively, these data indicate dual mechanisms for fructose-mediated hepatic lipogenesis- one *via* activation of the transcriptional DNL program, and another by providing DNL substrates in the form of microbiome-derived acetate.

#### Microbiota as a Modulator of Hepatic Lipid Homeostasis *via* FXR and TGR5

Clues for the role of bile acids (BA) in TG synthesis came in the 1970s when administration of chenodeoxycholic acid (CDCA) for gallstones also resulted in reduced circulating TGs (46). Conversely, patients treated with BA sequestrants were found to have elevated hepatic and serum TGs and VLDL (47). Bile acid synthesis from cholesterol which occurs exclusively in the liver is mediated by two key enzymes- CYP7A1 and CYP8B1, which through a series of reactions catalyze the production of CDCA and cholic acid (CA) respectively (48). CDCA is further converted to α, then β-muricholic acid (β-MCA) in mouse livers and into ursodeoxycholic acid (UDCA) in human livers. These primary BAs are further conjugated in the liver to the amino acids taurine or glycine to generate conjugated BAs such as taurocholic acid (TCA), tauro-alpha/beta-muricholic acid (T-α/β-MCA), etc. (49). Primary BAs are then stored in the gallbladder, wherein they are released upon meal ingestion to facilitate absorption of nutrients across the small intestine (SI). Approximately, 95% of BAs are reabsorbed in the ileum and are acted upon by gut microbiota to undergo de-conjugation by the bacterial enzyme bile acid hydrolase (BSH) and further dehydroxylation by bacterial dehydroxylases to generate the secondary BAs lithocholic acid (LCA) and deoxycholic acid (DCA) from CDCA and CA, respectively (50). Therefore, the gut microbiota plays a key role in maintaining BA composition, and will likely be impacted by any perturbations in microbiome composition.

The Farnesoid X-receptor (FXR) is a ubiquitously expressed nuclear receptor (NR), and plays a particularly important role in gut-liver signaling. Like most NRs, FXR has a N terminal ligand-independent activation function (AF1), a highly conserved DNA-binding domain (DBD), a ligand binding domain (LBD), and finally a C-terminal ligand-dependent activation function (AF2) (51). FXR forms a heterodimer with retinoid X-receptor (RXR), and when there is no ligand binding, the FXR-RXR heterodimer remains bound to FXR responsive elements (FXREs) within the promoters of FXR target genes, bound to co-repressors (52). Upon ligand-induced activation, co-repressors leave the FXR-RXR heterodimer to make way for co-activators, thus upregulating target gene transcription (52). While FXR was initially found to be weakly activated by farnesoid, an intermediate of mevalonate metabolism, it was later found that despite low affinity, BAs potently activate FXR in the order of CDCA > LCA = DCA > CA (53). BA binding to FXR in the intestine leads to the secretion of FGF15 in mice and FGF19 in humans into the hepatic portal vein (54, 55). Upon reaching the liver, FGF15/19 bind to their receptor FGF4 resulting in down-regulation of CYP7A1 and CYP8B1 to stop BA synthesis (54, 55). In this fashion, FXR tightly regulates BA production in the liver.

The role of the beneficial effects of FXR on glucose and lipid metabolism has been studied extensively across many mouse models (56–59). In brief, FXR activation reduces DNL by suppressing the transcription of Srebp-1c (60). It increases TG degradation by inducing the expression of PPARα and fibroblast growth factor (FGF) 21, both activators of fatty acid oxidation (61). Lastly, FXR promotes TG hydrolysis by increasing the expression of apolipoprotein (Apo)-CII which is an activator of lipoprotein lipase (LPL) (59). Taken together, FXR reduces hepatic steatosis by reducing DNL, increasing fatty acid oxidation, and increasing TG clearance.

To elucidate the role of the microbiome in FXR signaling, Jiang et al. treated mice with antibiotics and analyzed changes in BAs and progression of fatty liver (62). Microbiome depletion led to significant increases in the levels of T-β-MCA and TCA, as the bacterial enzyme BSH that catalyzes the conversion of T-β-MCA to MCA is missing in antibiotic treated mice. Increased levels of T-β-MCA inhibits intestinal FXR, which, in turn, reduces the transcription of ceramide synthesis-related genes, resulting in reduced levels of ceramide (62). Since ceramide regulates the cleavage and maturation of the pro-lipogenic Srebp-1c, there is a resultant reduction in HFD-induced hepatic DNL upon antibiotic treatment. Therefore, HFD-feeding leads to increased conversion of T-β-MCA to MCA by the gut microbiome, activation of intestinal FXR, followed by an increase in ceramide synthesis, which upon reaching the liver cleaves Srebp-1c to its active form, thereby increasing hepatic DNL (62) (**Figure 1**).

Several human studies report that both primary and secondary BAs are elevated in patients with NAFLD (63–65). Part of the explanation for this was the increased abundance of the taurine and glycine de-conjugating bacteria *Escherichia* and *Bilophila*, which catalyze the conversion of CA to the secondary BA DCA, which is antagonistic to FXR (63). Due to this inhibition of intestinal FXR, there was reduced secretion of FGF19, thus disrupting the feedback loop and maintaining elevated levels of CYP7A1 and CYP8B1 (63). As a result, there is continued production of BAs in patients with NAFLD, increasing the total primary BA pool size. In another study, NAFLD patients were found to have reduced levels of hepatic FXR, increased cleavage of Srebp1-c, and significantly higher hepatic TGs. Collectively, changes in gut microbiome composition in NAFLD contributes to disrupted primary and secondary BA production, reduced FXR signaling, and resultant fatty liver. Indeed, recent clinical trials have demonstrated that synthetic FXR agonists such as obeticholic acid have a beneficial effect in patients with NASH (66). Additional studies with a larger sample size need to be conducted to validate these findings.

Takeda G protein-coupled Receptor 5 (TGR5) is a G-protein coupled receptor which is less abundant than FXR, but is still highly expressed in the gallbladder, ileum, colon, and on hepatic macrophages known as Kupffer cells (53). As a GPCR, TGR5 activation leads to increase in cyclic AMP levels, thereby increasing the expression of protein kinase A which further mediates downstream effects. BAs activate TGR5 in the order of LCA > DCA > CDCA > CA, implying that TGR5 signaling is strongly associated with the microbiome as both LCA and DCA are products of the microbiome (53). TGR5s key role in the intestine is to facilitate secretion of the incretin hormone GLP-1 from enteroendocrine cells therefore increasing the secretion of insulin from pancreatic beta cells (67). In addition to imparting other metabolic benefits, the administration of GLP-1 agonists in *ob/ob* mice significantly reduced hepatic steatosis, both by driving down DNL, and up-regulating fatty acid oxidation (68) (**Figure 2**). Clinical trials with TGR5 agonists are currently underway for the treatment of NASH, and hold promise due to TGR5’s influence on GLP-1 signaling.

### Changes in the Gut Microbiome Disrupt Gut Barrier Function

The gut barrier is the first line of defense between intestinal luminal contents and circulation, and mostly consists of the epithelial barrier and the over-laying mucus layer. The epithelial barrier consists of a monolayer of adjacently aligned epithelial cells, a vast majority of which are enterocytes/colonocytes. This layer is also interspersed with four other epithelial cell types—goblet cells, enteroendocrine cells, Paneth cells, and microfold cells (69). Underneath the epithelial cell monolayer is the lamina propria, which houses innate and adaptive immune cells such as T cells, B cells, macrophages, and dendritic cells (70). Finally, under the lamina propria lies a vascular network that eventually converges into the portal vein which, in turn, empties into the liver.

Goblets cells are specialized mucus secreting cells embedded within the epithelial monolayer (71). Secreted mucus is composed of glycosylated mucin proteins that form a gel-like layer and sit above the epithelial monolayer (71). The small intestine (SI) and colon have very distinct physiologies (72). The SI has Immunoglobulin As (IgA) and anti-microbial peptides (AMPs) which are secreted into the mucus layer by plasma cells within the lamina propria, and Paneth cells respectively, making the SI relatively less hospitable for bacterial growth (73, 74). Compared to the SI, the colon has a significantly greater number of goblet cells, and hence more mucus. Unlike the SI, the colon has two layers of mucus, with the bottom layer sitting right above the epithelial monolayer, and is more “tight” in consistency (72). A “loose” mucus layer overlays the bottom layer. This outer mucus layer serves as a habitat for colonic gut microbes (72). Since the colon has fewer Paneth cells, there is less IgA and AMP secretion, which in combination with more mucus production and thickness, makes it a more fertile ground for gut microbes (72). Owing to these differences between the SI and colon, gut microbiome composition varies along the gastrointestinal tract (GI) as well, with more aerobic and facultative anaerobes in the duodenum and jejunum, and more fiber-fermenting, bile acid-metabolizing anaerobes in the colon.

Under the mucus layer lies the intestinal epithelial monolayer. Transport of molecules between the intestinal lumen and the underlying vascular layer is regulated by junctional complexes between epithelial cells within the monolayer (71). The three most important junctional complexes are tight junctions (TJs), adherens junctions (AJs), and gap junctions (75). TJs include proteins like zona occludin-1 (ZO-1), occludin, and members of the claudin family which seal intercellular space. AJs are found below TJs, and along with gap junctions, they help maintain the integrity of the epithelial monolayer and facilitate cell-cell communication. Intracellularly, TJs and AJs are attached to actin and myosin, thereby playing important roles in cytoskeletal dynamics. It should be noted that the gut barrier is not a static organ, but is actually rather dynamic and sensitive to changes occurring in the gut (75).

**Figure 3:**
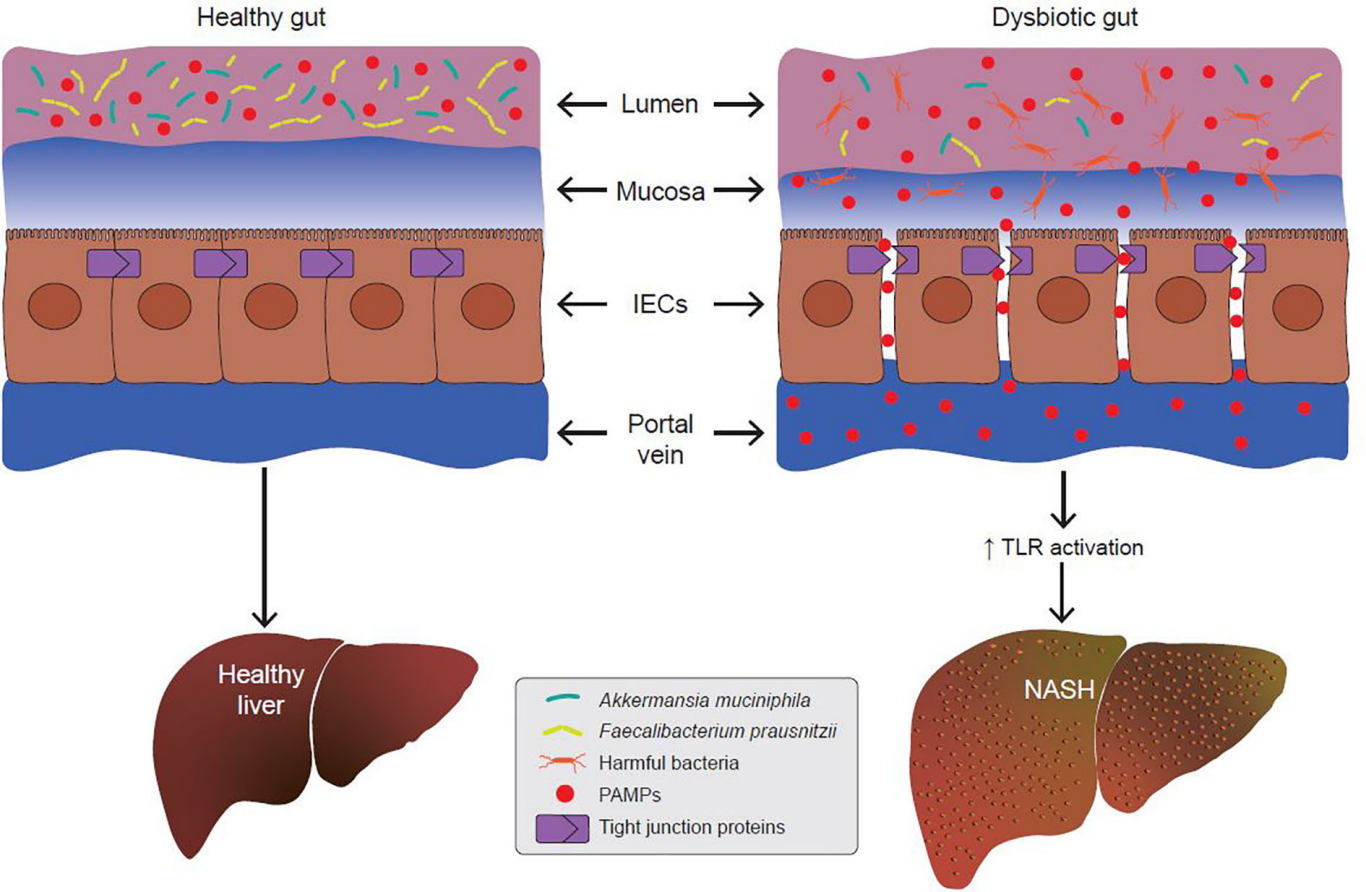
A dysbiotic gut promotes the development of non-alcoholic steatohepatitis (NASH). In metabolic gut dysbiosis, the populations of beneficial microbes like *Akkermansia muciniphila* and *Faecalibacterium prausnitzii* decline, and the populations of harmful bacteria increase. Via various mechanisms, this results in disruption of tight junction proteins (TJPs) between adjacent epithelial cells. This allows for increased paracellular passage of pathogen-associated molecular patterns (PAMPs) into the portal vein. PAMPs are endogenous ligands for Toll-like receptors (TLRs), and their binding to hepatic TLRs results in activation of pro-inflammatory and pro-fibrotic cascades which promotes the development of NASH.

Since the gut barrier serves to keep intestinal luminal contents from entering the underlying vascular network, any disruption in its integrity leads to a condition called the “leaky gut”. Under certain conditions, expression of TJPs is reduced leading to increased permeability between adjacent epithelial cells. Increased paracellular permeability gives luminal contents access to the underlying lamina propria and vascular network. Leakage of bacterial antigens into the vascular network, portal vein and liver leads to increased hepatic inflammation due to activation of immune signaling (76–78) (**Figure 3**). Indeed, metabolic diseases are often associated with a loss of intestinal barrier function and an increase in passive transport of microbial pathogen associated molecular patterns (PAMPs) into the body (79) (**Figure 3**). Emerging evidence suggests a link between a dysfunctional gut barrier and human NAFLD (80–82). A meta-analysis based on five clinical studies demonstrates a linear relationship between increased gut permeability and NAFLD progression, with stronger correlations as disease severity increases (82). Specifically, 39.1% of NAFLD patients had displayed increased intestinal permeability versus only 6.8% of HCs. In addition, it was found that patients with NASH were more likely to have this phenotype with the incidence of gut permeability in this subgroup being 49.2% higher compared to patients with NAFLD as a whole. These data suggest that inflammatory events occurring in the pathophysiology of NASH might be a function of increased gut permeability.

To demonstrate that increased intestinal permeability precedes NASH, Mouries et al. showed that intestinal epithelial barrier (IEB) disruption in mice occurs within 48 h of HFD-feeding (83). This was evidenced by reduced expression of ZO-1 and increased bacterial translocation into the ileum and cecum lamina propria. Plasmalemma vesicle-associated protein 1 (PV1), a marker for gut vascular barrier (GVB) damage was unchanged at 48 h. Following 1 week of HFD-feeding, PV1 expression and intestinal permeability were significantly upregulated along all sections of the gut, and stayed that way until the end of the 24-week study. In both 1-week and 6-week HFD-fed mice, disruptions in the IEB and GVB preceded signs of hepatic steatosis and IR, indicating that these are early events in the development of NASH. Impaired GVB is then maintained through development of IR and inflammatory NASH. To further support the hypothesis that a dysbiotic gut disrupts epithelial barrier integrity, when SPF mice were transplanted with fecal matter from control diet and HFD-fed mice, mice receiving FMT from HFD-fed mice had increased adipose mass and expression of PV1, suggesting that HFD-feeding induces dysbiosis, which disrupts the GVB, which, in turn, correlates with increased intestinal blood vessel permeability. Collectively, these data suggest a linear sequence of events- IEB disruption, GVB disruption, IR and hepatic steatosis, and finally NASH (83).

In contrast to the above study, Thaiss et al. absolved the gut microbiome of any culpability in IR-driven gut permeability (84). In addition to showing increased gut permeability in *db/db* and *ob/ob* mice, the authors were able to show similar gut barrier dysfunction in STZ-treated mice, therefore demonstrating that IR-driven gut barrier perturbations are associated with, but do not require obesity. To determine the consequence of barrier dysfunction, the authors used a bioluminescent variant of *Citrobacter Rodentium* to track infection *in vivo*, which mimics human enteropathogenic *E. coli* infections. In addition to being hyperglycemic, STZ-treated mice exhibited reduced expression of ZO-1 along with increased gut permeability. Upon receiving *C. rodentium*, these mice showed increased susceptibility to infection and systemic translocation, enhanced bacterial growth, epithelial adherence and systemic spread. To determine whether these gut dysfunction signals were arising from microbiome alterations upon STZ treatment, FMTs were performed with feces from STZ-untreated and treated mice. No gut barrier dysfunction and associated increase in bacterial infection was seen in mice receiving FMTs from STZ-treated mice, demonstrating that the gut barrier dysfunction observed in these mice is independent of the microbiome. RNA sequencing of IECs revealed global reprogramming of the epithelial transcriptome of STZ-treated mice. In particular, it was found that the transcription of the GLUT2 gene which is responsible for glucose uptake in IECs was significantly upregulated in STZ-treated mice. IEC specific GLUT2 knockouts did not show increased permeability, reduced TJPs or increased susceptibility to infection upon STZ treatment. Taken together, these data suggested that IR-driven gut barrier dysfunction is independent of changes in the gut microbiome, and instead is dependent on GLUT2 dependent signaling in IECs (84).

A key feature that sets the above study apart from the work of Mouries et al. is the animal model used. STZ injection is typically representative of Type 1 (T1D), while *ob/ob*, *db/db*, and HFD-feeding models are more representative of Type 2 diabetes (T2D. In humans however, T1D, much like T2D is often accompanied by the presence of metabolic syndrome, thereby making it challenging to investigate microbiome-independent mechanisms behind gut barrier dysfunction in human T1D (85). In conclusion, in mouse models of diabetes, gut barrier dysfunction in T1D is driven by GLUT2 signaling in IECs and in T2D is driven by disruptions in the gut microbiome, and precedes the development of NASH.

Much like NAFLD/NASH, diabetes also has been linked with gut dysbiosis and barrier dysfunction. For example, examination of 345 patients microbiome samples demonstrated a reduction in butyrate producers and increase in opportunistic pathogens in the diabetic microbiome (86). Another study confirmed a significant reduction in the population of *Bifidobacteria* and *Verrucomicrobia* (87). Specifically, *Akkermansia muciniphila* of the *Verrucomicrobia* phylum has been shown to be significantly reduced in diabetes in both mouse and human studies (87–89). Indeed, oral administration of *A. muciniphila* resulted in marked improvements in metabolic parameters in genetic and diet-induced models of diabetes, positioning it as a beneficial microbe (89). It is most abundantly found in the loose outer mucus layer of the colon, and uses polysaccharides in the mucus as substrates to generate SCFAs like acetate and propionate (90). *A. muciniphila* supplementation was reported to restore the colonic mucus layer to its normal thickness in HFD-fed mice, the mechanisms behind which remain unclear (89). In another report, pasteurized *A. muciniphila* and Amuc_1100, the pili protein in *A. muciniphila*, were shown to improve gut barrier integrity by upregulating the expression of some TJ proteins (91). Perhaps, most strikingly, *A. muciniphila* supplementation was shown to significantly reduce circulating LPS levels, which suggests that it lead to improvements in gut barrier integrity (89, 91). In the strongest case yet for using *A. muciniphila* supplementation as therapy for gut-related and hepatic pathologies, a small exploratory proof-of-concept study was conducted on 40 obese male and female individuals (92). Participants were divided into three groups- placebo, pasteurized *A. muciniphila* and live *A. muciniphila* treated groups. At the 3-week end-point, in addition to demonstrating no adverse responses associated with *A. muciniphila* supplementation, the group receiving pasteurized *A. muciniphila* had modest, yet statistically significant, reductions in circulating LPS, AST and ALT levels (92). Although more studies with larger patient cohorts are required to confirm these findings, the use of *A. muciniphila* as a therapeutic agent still holds promise.

Another important function of *A. muciniphila* is its ability to support the growth of butyrate producing bacteria by a method known as cross-feeding (93). More specifically, in using mucus as a substrate, *A. muciniphila* produces the SCFA’s acetate and propionate, which are, in turn, utilized by bacteria such as *Faecalibacterium prausnitzii*, which produce butyrate (93). Much like *A. muciniphila*, loss of *F. prausnitzii* also correlates with development of T2D (94) (**Figure 2**). By producing butyrate, *F. prausnitzii* enhances mitochondrial function in colonocytes, thereby stabilizing HIF-1α in the gut (95). HIF-1α although considered “bad” in other contexts, has been shown to improve gut barrier integrity through yet unclear mechanisms. In addition to maintaining hypoxic conditions in the gut, butyrate supplementation to colonocyte and epithelial cell lines has led to increased transepithelial resistance (TEER), marking improved barrier function (96). Most importantly, one study found that *F. prausnitzii* supplementation in HFD-fed mice led to a significant reduction in diet induced steatosis, ALT and AST levels, thereby suggesting that increased butyrate production improves barrier integrity and consequently improves NASH (97).

While the above data paints *A. muciniphila* as a good player, another study showed that consuming diets depleted of fiber led to significant proliferation of *A. muciniphila*, correlating with a significant reduction in colonic mucus thickness and a compromised gut barrier (28, 98). While it is unclear why *A. muciniphila* appears to be a negative component of the microbiome in this study, it is possible that consuming a diet low in fiber deprives *A. muciniphila* and associated cross-feeders of classic substrates. A known mucus degrader, it is possible that *A. muciniphila* instead shifts its metabolism to use mucus as a substrate, thereby feeding into a cycle of mucus consumption, reduction in mucus thickness and increased proliferation. Eventually, when mucus consumption exceeds production, a scarcity of substrate availability results, and the population of *A. muciniphila* declines. It is possible then, that reduced *A. muciniphila* population size in diabetic patients is a consequence, rather than cause of compromised gut integrity. This might also be the reason why pasteurized forms of *A. muciniphila* show an improvement in metabolic endpoints, because this form does not have mucus degrading activity. A prospective study where *A. muciniphila* populations are measured from the onset to full- fledged development of diabetes might be able to answer some of these questions, but until then, the jury is out on the role of *A. muciniphila* in gut barrier integrity.

### Changes in the Gut Microbiome Promote Progression of NAFLD

The previous section elucidates how gut microbiome dysbiosis occurring during metabolic syndromes can alter intestinal biology to make the gut more permeable. This allows passive transport of microbial PAMPs from the intestinal lumen into the portal vein, and eventually the liver (**Figure 2**). Before diving into how PAMPs contribute to NASH, it is first important to appreciate its pathophysiology. As mentioned, the first step of NAFLD is almost always the development of fatty liver. The second step involves multiple hits like IR, increased gut permeability, inflammation, and reactive oxygen species (ROS) production which leads to the progression of a more inflammatory, fibrotic NASH phenotype. Progression of fatty liver to fibrosis affects all liver cell types (99). While hepatocytes appear injured and undergo a form of cell death termed apoptosis, the resident liver macrophages, Kupffer cells (KCs), start secreting pro-inflammatory chemokines and cytokines. Finally, quiescent stellate cells (SCs), which are the major storage site for retinoids, are activated (99). Activation of stellate cells leads to loss of retinoids and increased expression of signaling receptors including the transforming growth factor β (TGF-β) receptor. Activated SCs proliferate and secrete extracellular matrix proteins to form a fibrous scar, which imparts a “fibrotic” phenotype to NASH (99). PAMP receptors such as the toll-like and nod-like receptors (TLRs and NLRs) are expressed on the cell surface of hepatocytes, Kupffer cells (KCs), and stellate cells (SCs), and are known to contribute to the inflammatory and fibrotic phenotype of NASH (**Figure 2**). While suppressed in healthy liver, TLR signaling is activated in the presence of pathogenic microorganisms and bacteria-derived molecules. Since other reports have already reviewed the role of TLRs in NAFLD in great detail, this section will briefly highlight some of the major findings (100, 101).

Of all the TLRs, TLR2, -4, -5, and -9 have been shown to contribute to the inflammatory and fibrotic signaling that characterizes NASH. In hepatocytes, LPS binding to TLR4 recruits MyD88, an adapter protein, which, in turn, leads to the activation of nuclear factor kappa-light-chain-enhancer of activated B cells **(**NF-κB) and mitogen-activated protein kinase (MAPK) signaling pathways (102). NF-κB is a transcription factor which upregulates the transcription of pro-inflammatory cytokines including interleukin (IL)-1, 2, 6, and 8 (103). In addition to its role in increasing hepatocyte inflammation, TLR4 plays a role in KC and SC crosstalk. LPS binding to TLR4 in SCs leads to increased production of adhesion molecules and chemokines like vascular cell adhesion protein (VCAM) and methyl-accepting chemotaxis protein (MCP) (104, 105). Adhesion molecules attract KCs and these recruited KCs secrete the pro-fibrogenic TGF-β which binds to TGF-β receptors on SCs (104, 105). This stimulates the secretion of collagen from SCs into hepatocytes, marking the beginning of liver fibrosis. Indeed, KC-specific knockdown of TLR4 in mice on a methionine choline-deficient (MCD) diet led to a significant reduction in hepatic TGs, reduced expression of inflammatory and fibrosis markers, and a resultant reduction in histological markers of NASH (106). Similar findings, demonstrating increased TLR4 mediated signaling contributing to NASH development, have been reported by other groups (106, 107).

In addition to TLR4, other TLRs mentioned in the paragraph above also have been implicated in NAFLD, but there are only a handful of reports elucidating their roles. TLR2 is expressed by HSCs and KCs and is a receptor for bacterial peptidoglycan. To investigate the role of TLR2 in hepatic inflammation, Miura et al. treated KCs with a synthetic TLR2 ligand Pam_3_CK_4,_ and an endogenous ligand palmitic acid (PA) (108). While priming with Pam_3_CK_4_ alone was enough to increase the expression of NLRP3, IL-1β and IL-1α, caspase-1 activity was only induced when KCs were treated with PA after priming with Pam_3_CK_3_ first, indicating that both signals were required for activation of the inflammasome complex (108). This further led to the cleavage of the pro-inflammatory cytokines IL-1β and IL-1α to their active form, thereby upregulating hepatic inflammation (108). On the other hand, knock-out of TLR2 has yielded conflicting results in different mouse models, with the more conventional metabolic syndrome models suggesting that loss of TLR2 is protective against NASH (108–110). TLR5 is expressed in hepatocytes, and is a receptor for bacterial flagellin. While the exact role of TLR5 in NAFLD remains unknown, two separate studies have shown that knock-down of TLR5 accelerates hepatic steatosis, susceptibility to liver injury and NASH, thereby assigning it a more protective rather than harmful role (111, 112). Finally, TLR9 is expressed in Kupffer cells, and is a receptor for bacterial DNA. TLR9 activation signals through NF-κB to increase the expression of the cytokine IL-1β from KCs, and induces chemotaxis of macrophages and neutrophils, thereby leading to hepatic steatosis, inflammation and fibrosis (113, 114).

In conclusion, with the exception of TLR5, hepatic TLRs, upon binding by gut bacteria-derived products, set into motion a cascade of inflammatory and fibrotic signals, thereby abetting the progression of fatty liver to NASH.

## Discussion

Increasingly, alterations in the gut microbiome have been correlated with NAFLD progression. This remains an important discussion in microbiome research relating to NAFLD for several reasons. Firstly, a unique microbial signature associated with the different phenotypes within NAFLD could serve as a non-invasive tool for accurately determining severity of disease. Secondly, predicting disease progression and prognosis will be easier and less invasive, in comparison to performing a liver biopsy each time an individual comes into the clinic for follow-up. Thirdly, a unique microbial profile in NAFLD overlaid with metagenomic signatures will help predict host metabolic responses, leading to more personalized interventional approaches. Lastly, therapeutically shifting a “disease promoting” microbiome to an “anti-NAFLD/NASH” microbiome remains an attractive strategy for thwarting or reversing the course of NAFLD progression.

As far as host metabolism is concerned, the microbiome may contribute to both hits of NAFLD; first by promoting development of fatty liver *via* DNL, then through hepatic TLR activation due to dysbiosis (**Figure 1**). Use of microbial-derived acetate as a substrate for hepatic DNL is a particularly striking finding, as thus far, the hepatic transcriptional lipogenic program alone was thought to play a role in DNL. The studies described in this report identified microbial populations that produce SCFAs, but there are still limited data on specific acetate producers. The modest reduction of fecal acetate levels as fatty liver progresses to NASH also is an important finding because this throws additional light on microbiome-dependent pathophysiology of NAFLD. Do acetate producing microbiome populations decline as NAFLD progresses? What are these populations and could their decline potentially predict onset of fibrosis? Many such outstanding questions remain.

This report also reviewed literature that investigated dysbiosis-induced increases in gut permeability in metabolic syndrome including NAFLD. Interestingly, T2D associated increases in gut permeability were found to be microbiome-dependent, while T1D associated increases in gut permeability relied more on glucose transport pathways in IECs. This is hardly surprising because the pathophysiologies of both are fairly independent, and their gut microbiome signatures are different as well. T2D-associated increases in gut permeability were found to be inversely correlated with *A. muciniphila* and *F. prausnitzii* populations. While the literature has painted *A. muciniphila* as a beneficial microbe, we are of the opinion that its reduced populations are a result, and not cause of T2D induced gut barrier perturbations, given its mucin degrading activity. Given that *A. muciniphila* cross feeds *F. prausnitzii*, a decline in the population of the former adversely affects the latter. Since *F. prausnitzii* is a key butyrate producer, its loss logically leads to compromised gut barrier function. Further research on the full spectrum of functions of both microbes are required before making any firm conclusions about their applicability in human disease.

Lastly, increased barrier permeability leads to leakage of luminal LPS into the portal vein and liver, leading to activation of hepatic TLRs and NASH (**Figure 3**). Our report described numerous studies that characterized the pro-inflammatory and fibrogenic role of different TLRs in NASH. Targeting circulating LPS and TLRs might be important therapeutic avenues for the treatment of NASH.

As far as translational application of the microbiome in the treatment of NAFLD is concerned, a clinical trial is currently underway that aims at repopulating the gut microbiome of NASH patients *via* FMT from lean donors (ClinicalTrials.gov Identifier: NCT02469272). The primary end-point of the study at the end of the 12-week FMT period is degree of hepatic steatosis as determined by MRI. The secondary end points are liver function tests and markers of insulin sensitivity. Data from this study will provide preliminary clues on the safety, viability, and efficacy of the use of FMT for the treatment of NASH. Follow-up large-scale studies will be required to truly validate any beneficial findings before FMT is considered as a therapeutic intervention for NASH.

## Author Contributions

KJ and TC contributed to the writing of this manuscript. All authors contributed to the article and approved the submitted version.

## Conflict of Interest

All authors are employees of AstraZeneca, and may hold stock in AstraZeneca. This work was fully funded by AstraZeneca. The funder has no role in the design, writing or decision to publish this review manuscript.
